# "The Most Effective Experience was a Flexible and Creative Attitude"—Reflections on Those Aspects of Spiritual Care that were Lost, Gained, or Deemed Ineffective during the Pandemic

**DOI:** 10.1177/1542305020987991

**Published:** 2021-04

**Authors:** Anne Vandenhoeck, Cheryl Holmes, Cate Michelle Desjardins, Joost Verhoef

**Affiliations:** KU Leuven, Leuven, Belgium; Spiritual Health Association, Melbourne, Australia; Transforming Chaplaincy, Chicago, IL, USA; OLVG, Amsterdam, the Netherlands

**Keywords:** Chaplaincy during Covid-19 pandemic, lost ways of spiritual care, new ways of spiritual care

## Abstract

This paper presents and discusses data from three of the qualitative questions in the international COVID-19 survey: What was the most important aspect of spiritual care that was lost during the pandemic? What was new to you during this pandemic? What are the new ways of delivering spiritual care you have experienced? Of these new experiences, what do you think was the most effective?

## Introduction

Over many decades chaplains from across the globe have been in discussion about models of spiritual care and recognition as healthcare professionals (Cadge, 2019; Swinton, 2013; Timmins et al., 2017). The advent of the SARS-Cov-19 pandemic provides an opportunity to once again shine a light on the practices and professionalism of chaplains. A mixed methods survey was distributed to chaplains across the USA, Europe and Australia to explore their experiences during the pandemic with 1657 responses received. The research study was approved by the Ethics Review Board of the KU Leuven, Belgium (G-2020-1964-R2). This paper presents and discusses data from three of the qualitative questions:

1. What was the most important aspect of spiritual care that was lost during the pandemic?
2. What was new to you during this pandemic? What are the new ways of delivering spiritual care you have experienced?
3. Of these new experiences, what do you think was the most effective, and why?

In order to discuss the answers to the qualitative questions, two steps were taken. First all the answers in other languages were translated to English. Then thematic analysis was applied to identify and interpret patterns of meaning.

### The Most Important Aspect of Spiritual Care that was Lost during the Pandemic

When people experience loss, chaplains give them space to talk about it. Therefore, it seemed appropriate in the worldwide survey on Covid-19 to ask chaplains what their losses were. What did they see disappear from their pre-pandemic chaplaincy? For some chaplains everything was lost. They were made redundant or sat at home. For others nothing was lost. On the contrary, they gained visibility and assignments. Most chaplains though found themselves in between these extremes. The responses to the question what most important aspects of spiritual care were lost, can be divided into different categories: loss of physical presence or support and touch, of shared group and community moments, of not being able to work as we should as health care professionals, of a feeling of safety, of working with volunteers, of a backup theology.

#### The Loss of being Present and of Touch

The majority of chaplains worldwide named the loss of presence (being there)^1^ and touch as the most important aspect of spiritual care that was lost. Even when allowed to visit patients, chaplains needed to wear masks and thus spiritual care became ‘faceless’.^2^ Traditionally, a lot of emphasis is placed on being present, building trust, being connected as part of the identity and main competencies of the chaplain (Fallers Sullivan, 2014; Cadge, 2019).The pandemic has shaken this part of chaplaincy to the core. But the survey also shows that the professional identity of the chaplain sought to overturn losses to gains. In chaplaincy there is a tension between a personal, vocational identity, which emphasizes presence and spirituality, and a professional identity which is in the process of being developed through research emphasizing skills and integration in healthcare. Chaplaincy is best served when both poles are present and chaplains make appropriate use of them. The distorted connectedness through absence of presence and touch was also noted in contacts with colleagues/staff and becomes even more painful when it concerns people who are challenged in communication because of dementia or other cognitive or physical disparities. Some chaplains reflect on the loss of the spontaneous, casual, incidental contacts with patients, families, staff. The pandemic made chaplains aware of the significant contribution to spiritual care of non-verbal expressions and touch. This is equally experienced in other healthcare professions but articulated in chaplaincy. Cultural differences and discussions about the ethics of permission regarding touch seem to have moved to the background during the pandemic. Masks and other protective material make it hard to read emotions and create distance. Telechaplaincy adds to not being able to use body language.

#### The Loss of Shared Spiritual Experiences with Patients and Families

Serious illness, end of life and death are likely to invoke the need for rituals and symbols (Pace & Mobley, 2016). Chaplains on such occasions bring people together and offer rituals or use symbols to say goodbyes, to remember, to mourn, to create transition, to connect with the sacred. In the survey participants express the loss of the meaningful marking of life events through rituals and symbols. Sacred spaces like chapels, interfaith rooms or prayer rooms remain closed or are used for other purposes. Candles cannot be lit, rituals are taking place at the bedside without family present, some perform rituals from a distance (for example a ritual in the chapel for a dying patient in a hospital room), sacraments cannot be given. It stressed the loneliness of people even more: “*The loneliness bereaved parents/families experienced when they could not attend ritual/service for their deceased baby*.” (participant 2551 Europe). The connectedness with others and the sacred cannot be expressed through shared rituals, sacraments, prayer or the use of symbols. Through the lack of rituals, a loss of religiosity was experienced: “*Priests were not permitted to bring communion to our Catholic residents/clients. This left a hole for many of our people at a time when they needed this.*” (participant 1160 North America). Chaplains also miss other community forming moments like singing together in services or with residents, discussion groups or groups that talk about meaning, meditation groups, prayer groups etc.

#### Not Being Able to Work as We Should as Healthcare Professionals

Chaplains expressed frustration, helplessness and sadness not being able to give the needed and appropriate end of life care to patients and families. Knowing patients are lonely, anxious and dying alone, causes moral stress to chaplains. “*People died alone. We allowed that decision to be made. Maybe it was right… maybe it was wrong. It was complicated for sure and there were a lot of values at play. I feel like chaplains sat on the sidelines and allowed it*.” (Participant 1149, North America). Some chaplains expressed the fact that the demand for spiritual care exceeded the time and staffing available. Others felt deprived of their professional value by being less/not included in multidisciplinary teams, by the lack of collegial response, by the emphasis on medical care, by the lack of protective material for chaplains, by their roles being taken over by other healthcare professionals or by not being a priority in providing telehealth options. “*There was a lot of power-grabbing by other professions. Chaplaincy received tokenistic roles; treated as afterthought*.” (participant 615 Western Australia). Some participants expressed feeling invisible and lonely. The often limited communications with staff or team members – not being able to experience fellowship with teams – contributed to that. Being a professional was further limited by the often challenging technical aspects of communication with patients and residents. The fact that patients or residents needed help with devices had an impact on the confidentiality in the contact with the chaplains. Part of being a professional health care chaplain is discerning who might benefit from your support and taking the initiative to contact them. This reaching out was limited by the pandemic and efficient patient care was impacted when communication between team members became harder. Some participants refer to complicating factors in the context: working with less chaplains due to their health issues, no volunteers to support spiritual care as they often belong to the groups at risk, no support from outside faith representatives, double pressure because of implications of the pandemic at work and home. But the most referred to injury was seeing suffering and not being able to support. What makes it more potentially harmful is the comment of some participants that there was no time for selfcare or refill while having to deal with feelings of helplessness and witnessing inhumanity regarding elderly being deprived of seeing their loved ones or people dying without dignity.

#### A Loss of Feeling Safe

Among the answers were expressions of loss of trust and safety. Chaplains, like other health care professionals need to feel safe in order to fully function. The lack of protection material, the illusion of being invulnerable as staff, the fear of being at risk, the lack of adequate policies and protocols had the potential of partly paralyzing chaplains. Some chaplains were especially concerned of being spreaders of the virus when going from one patient to another.

#### Lack of Meaning and a Backup Theology

Chaplains have their own meaning system, tied to their personality and faith, which through confrontation with a pandemic brings forth the need to attribute meaning to suffering, loneliness and death. Existential questions are also experienced by chaplains. Where is God in all of this? A chaplain described the tension between a God of proximity and the lived reality in hospital rooms. Some chaplains describe a feeling of less connectedness with the traditional religious and spiritual resources. Their personal faith reflections inform their way of working and therefore need to be dealt with professionally. Other chaplains regret the lack of pastoral theological reflections on spiritual care during crisis times.

### What was New during the Pandemic?

Very few chaplains reported “no new ways” of doing chaplaincy during the pandemic. A few reported continuing to see patients in person or through specific services, such as a Hospital Crisis Team, that continued without changes.

#### Use of Several Digital Technologies

The use of digital technologies to connect with and provide care to patients, families, and/or staff was the primary new mode of providing chaplaincy described by participants. This was frequently coupled with “working remotely”, often from home, and losing access to office space or even being “barred” from hospital units. For some, adopting this technology involved “a huge learning curve” and a great deal of loss, as reported above. “During the pandemic I switched to providing spiritual care almost exclusively over the phone to families, which is the inverse of how I was spending the bulk of my time before the pandemic” (participant 177, North America).

The most frequently reported technology utilized was the telephone. Many chaplains reported learning and using a variety of different technologies at the same time, including Zoom, Facetime, Whatsapp, Microsoft Teams, Skype, and Webex. Participants also reported using iPads to help facilitate their conversations with patients or to help facilitate conversation between patients and their families who remained at a distance. While many chaplains expressed loss and grief over moving to distanced, technology-mediated care, others acknowledged the possibilities it afforded. *“The technology had the potential to expand rather than contract our reach during the crisis, since we could provide care to patients from whose rooms we were otherwise barred”* (Participant 241, North America).

The use of these digital technologies went beyond one-on-one interactions and included setting up telephone hotlines, for patients or for staff, and special prayer lines. Sometimes a chaplain ‘staffed’ the hotline or prayer lines, and other times the line had a recorded encouragement, prayer, or meditation. Further, with the loss of holding in-person services in many settings due to social distancing restrictions, chaplains employed video streaming or pre-recorded video to provide either religious services or meditation content, such as “televising daily meditation/reflection from the hospital chapel via 'Chapel Chanel’.

Chaplains also reported using these digital technologies, including video services, to support patient and family distinct religious and ritual needs. These included “conducting Baptisms without family present via video”, providing online Passover resources, and attending to other diverse religious needs: “We filmed a virtual Easter service. We coordinated sterilizable Ramadan blessings and a virtual Eid service” (participant 181, North America).

#### Novel Digital and Print Communications

Chaplains reported beginning to send newsletters, both print and through e-mail, to staff or to patients (especially in long-term-care settings). Sometimes these newsletters directly replaced worship services, such as “an Easter newsletter to all patients, since there couldn't be any worship services” Other times, particularly newsletters sent to staff, they focused on encouraging and spiritual content. Other chaplains found handwriting cards, notes, and letters to be an important way of connecting during the Pandemic. *“I rediscovered writing letters to connect with people in the nursing home.”* (participant 222, Europe) Some chaplains reported curating physical items and “kits” for spiritual encouragement to replace in-person chaplaincy care.* “I created a 'support pack' to have passed to patients I could not visit, either at that moment or at all”* (participant 231, Europe).

#### The Shift to Staff Support

One of the new aspects of chaplaincy care during the Pandemic was the “significant shift from patient care to staff support”, at times as a replacement for in-person patient care that was no longer possible, and at times primarily due to “increased referrals for staff support.” Sometimes this accompanied a sense of new and deeper connection to staff: *“Staff clearly felt more able/free to speak to chaplains about their concerns”* (participant 248, Europe). Staff support that respected restrictions around Covid included creation of “wellbeing hubs”, meditation spaces, hosting online support groups, and chaplains increasing the amount of time they were physically present with staff on units to support them.

#### New Forms of Prayer

Prayer is a central part of the chaplaincy role and profession (Massey et al., 2015). Modes of prayer shifted significantly for many chaplains during the Pandemic. Several new modes included “Praying in the hallways and not in rooms.” One chaplain wrote: “I began the practice of walking through the unit and praying at each window because I was not allowed in rooms” (participant 120, North America). Chaplains reported shifting from group prayer gathering to individual prayers, both themselves praying via Zoom or other digital technologies with patients, families, or staff, or facilitation such as “facilitating Catholic prayers over the phone due to no priests in the hospital.” Other chaplains reported continuing to pray with families but “praying with families outside of hospital” due to visitor restrictions.

#### Chaplains as Intermediaries

In light of many institutions increased restrictions on visiting loved ones who were in hospital either for Covid or other reasons, chaplains took on a new role of mediator between family and loved ones, and “supporting patients and families that were sepearated (*sic*)”. At times this facilitation and support brought forth creativity: “We made a digital photo archieve (sic): 3 times a week we took photos of ICU patients and send it to their family” (participant 205, Europe). In some cases, increased visitor restrictions required chaplains to go beyond a mediating or facilitating role into an advocacy role. Chaplains report *“advocating to ‘bend the rules’ to allow for families and loved ones to visit dying patients.”* (participant 247, North America)

#### End-of-Life Care during the Pandemic

Chaplains have a significant role to play in End-of-Life care in diverse healthcare settings (Flannelly et al., 2012). New forms of end-of-life and bereavement care reported during the pandemic included increasing chaplain-provided blessings for the deceased in the absence of ability for families to hold funerals, holding digital or video-streamed funerals, “Knitting hearts for dying patients/relatives” (participant 220, Europe) and “prayer during transfer of bodies from hospital morgue to Medical Examiner trucks” (participant 14, North America).

### Of These New Experiences, What do You Think was the Most Effective and Why?

Having described the new ways of delivering spiritual care experienced during the pandemic, participants were asked which were most effective and why. Responses ranged from the enthusiastic “All have been effective” to the dispirited “All them felt failures”. However, given the focus of the question, the majority of the 1126 responses described those experiences that were effective. Chaplains have always responded to the needs before them as they arise. It is work that is unpredictable and requires a high capacity for paying attention, assessing, planning and preparing. It is not surprising that this need for situational flexibility was highlighted during the pandemic. “The most effective experience was a flexible and creative attitude” (participant 955 North America).

Of the new experiences described, those using different forms of technology had the highest number of references and were nominated as most effective. Unsurprisingly new literature is already emerging on the effectiveness of telechaplaincy (Byrne & Nuzum, 2020; Sprik et al., 2020), although not all of the new forms of spiritual care included technology as was apparent in the section above. Having gained a clear picture of what was new, in this section we are interested to explore why these experiences were effective.

#### Increased Contact & Connection

Chaplains provide support to patients, families and staff (Timmins et al., 2018). While patients could be described as a captured audience, contact with families can be much more difficult to plan. Timing of visits may not coincide with the chaplain’s working hours or significant family members may be too far away to visit. With the advancement of telechaplaincy options, increased contact with family members was made possible and this was an experience many were keen to continue into the future. “The contact with family will continue with modification after the pandemic.” (participant 1546 Australia). Finding ways to connect with families who were unable to visit because of the pandemic restrictions highlighted the possibilities for connecting with families in the future who are unable to visit for other reasons. “More proactive contact with families outside of hospital. Use of online communication as a tool particularly when family/friends are distant or otherwise unable to visit.” (participant 793 North America).

Responses highlighted not just the opportunity for increased contact but the depth and quality of the contact. A number of participants reflected that this increased depth and quality may have been enabled because of the privacy of the online call, or peoples’ willingness to speak more openly without the face-to-face contact. “Honestly, the phone contact with family members was where most spiritual assessment occurred; a deeper connection forged than we sometimes get with patient's loved ones right there in the room.” (participant 616 North America).

Increased contact did not just occur between chaplains and families, but the new forms were effective because they enabled families to connect with their loved ones directly when they were unable to be in the room. “IPAD connection, bringing family contact into an isolated and lonely environment. Enabling family to be with each other in last hours”. (participant 580 Europe). Telechaplaincy also proved effective because it enabled the chaplains to have increased contact with patients. “Contacting patients by phone was effective. It allowed us to cover more patients than we could have otherwise”. (participant 1562 North America).

Closely related to the theme of contact, opportunities for increased connection were identified by many chaplains. The importance of connection or relationships is emphasized in many definitions and understandings of spirituality and it is therefore unsurprising that the chaplains reported that new experiences were effective because they enabled connection (Puchalski et al., 2014). In some cases, these connections were reported as being even better than pre-pandemic encounters with patients. “Phone support was/is actually really effective. In some ways I felt like I had better connections with patients because I was calling them at home in between treatments, instead of my usual of visiting them while getting chemo/infusions in the clinic” (participant 264 North America). Increased capacity to connect patients with their families was also assisted by the use of technologies. “Empowering families to use media platforms such as zoom to connect. Even before the pandemic many families struggled with distance and this tool became readily available to both connected and unconnected patients and families” (participant 1558 North America).

The move into using new forms of spiritual care also provided the impetus to introduce changes to practices that had previously been foreshadowed but had been met with resistance.

“We have been trying to increase connection to new admissions by encouraging pastoral care staff to phone patients/families that are unavailable when they visit in person. There has been a lot of resistance to this but now can be implemented as part of our regular admissions process.” (participant 614 North America)

Technology increased connection between teams and colleagues that were effective because they enabled more regular meetings, saved travel time and enhanced relationships. “Connecting with colleagues via skype was easy and saves travelling” (participant 1129 Europe).

#### Staff Support and Increased Awareness of Spiritual Care

As noted above, chaplains provide staff support as part of their role within health services. The pandemic provided increased opportunities to focus on staff support as staff struggled with the increased demands, pressures and workloads. Staff were also facing moral dilemmas and the risk to their own health and safety. “Caring for the staff became a very important task. We realised that the restructuring of the hospital had a large impact on their wellbeing. It still does. We got to be closer to the teams, more close than we already were” (participant 749 Europe). Staff support was seen to be effective because of the immediate benefit to staff wellbeing, but it also brought about increased awareness and understanding of spiritual care in the health services. Many reported on the positive impacts for the development of spiritual care services. “Realisation/appreciation of the value spiritual care services offers to staff (wellbeing), greater opportunities for promotion, service development, integration.” (participant 1523 Australia). Based on the effectiveness of providing spiritual care for staff many chaplains commented that this would continue to be a focus for the future. “Staff now understand much better what spiritual care providers can do for them, as well as for patients and families. This is something that will carry on after the crisis is over.” (participant 248 North America).

## Conclusion

It is clear from the abundance of written comments from chaplains that a lot was lost in spiritual care during the first wave of the pandemic in the first half of 2020. Some chaplains lost more than aspects of spiritual care, they lost their position. Most chaplains though missed the familiar practices of touch and presence, shared spiritual moments, a feeling of safety and meaning or supportive theological reflection. What seems to be most hurtful though was the loss of being able to give quality spiritual care to those who needed it most. The question about what was new in spiritual care during the pandemic in the first half of 2020 shows a strong resilience and capacity to adapt in chaplains worldwide. Chaplains started to work with the opportunities and technologies that could serve their spiritual care. They became creative with rituals, visits and prayer. They became advocates for patients and families. And they moved the ship of spiritual care even more into the direction of staff care when that became needed. Moreover, it becomes apparent that most chaplains have learned from their flexible attitude. They have developed new skills in the area of technology and the majority will keep working with technology as an addition to being physically present. They have learned that they can connect in a meaningful way with families, patients and staff online and reach people they would have otherwise not met. Finally they experienced that those new ways of working and providing staff care also integrated them more in whole person care and in the way health care institutions dealt with the crisis.

## Notes

1 Limited or no visits to non-Covid patients, no emergency calls, no house calls, no or limited visits to Covid-patients.

2 “Spiritual care became faceless. Even when we were with people, the masks prevented you from seeing each other. Also, touching: holding hands during prayer or for comfort or giving hugs. Isolation: being unable to be with people. families and staff.” (participant 1472 North America).
